# Extracellular matrix: an important regulator of cell functions and skeletal muscle development

**DOI:** 10.1186/s13578-021-00579-4

**Published:** 2021-03-31

**Authors:** Weiya Zhang, Yuan Liu, Hong Zhang

**Affiliations:** 1 Agricultural Technology Innovation Center in Mountainous Areas of Hebei Province and National Engineering Research Center for Agriculture in Northern Mountainous Areas, Hebei Agricultural University, No. 289 Lingyusi Street, Lianchi District, Baoding, 071001 Hebei People’s Republic of China; 2 Institute of Zoology, Chinese Academy of Sciences, Beichen West Road, Chaoyang District, Beijing, 100101 People’s Republic of China

**Keywords:** ECM, Skeletal muscle, Cell, Structure, Function, Application

## Abstract

Extracellular matrix (ECM) is a kind of connective tissue in the cell microenvironment, which is of great significance to tissue development. ECM in muscle fiber niche consists of three layers: the epimysium, the perimysium, and the endomysium (basal lamina). These three layers of connective tissue structure can not only maintain the morphology of skeletal muscle, but also play an important role in the physiological functions of muscle cells, such as the transmission of mechanical force, the regeneration of muscle fiber, and the formation of neuromuscular junction. In this paper, detailed discussions are made for the structure and key components of ECM in skeletal muscle tissue, the role of ECM in skeletal muscle development, and the application of ECM in biomedical engineering. This review will provide the reader with a comprehensive overview of ECM, as well as a comprehensive understanding of the structure, physiological function, and application of ECM in skeletal muscle tissue.

## Introduction

Skeletal muscle is an important organ of locomotion and metabolism in the body, which plays a very important role in maintaining the exercise balance, glucose metabolism [1], and energy metabolism [2] of the body. Muscle fibers and muscle progenitor cells (satellite cells) reside in the skeletal muscle microenvironment. The microenvironment, in which muscle fibers and satellite cells inhabit, also known as niche, has important effects on the growth of muscle fibers and myogenic differentiation of satellite cells. Extracellular matrix (ECM) presents in the muscle niche and is composed of proteins, polysaccharides [3], and RNA [4] etc., which plays an important role in maintaining homeostasis and regulating the development of skeletal muscle [5]. The ECM of skeletal muscle tissue contains three layers. The innermost structure is called the basal membrane (basal lamina), which supports and wraps a single muscle fiber. A number of muscle fibers form muscle bundles, which are wrapped by the perimysium. Moreover, a plurality of fasciculus form muscle mass, which are wrapped by the epimysium.

ECM is involved in skeletal muscle development from embryonic stage [6] to senescence [7]. Study showed that the excessive accumulation of ECM in the cell microenvironment of aging muscle inhibited the myogenic differentiation ability of satellite cells [8]. Researches indicated that the protein components in ECM participated in the myogenesis process of skeletal muscle progenitor cells, and the collagen secreted by satellite cells could maintain the quiescence of satellite cells [9–11]. Recently, Liu et al. confirmed that collagen I, a major ECM component, could promote the activation of focal adhesion kinase to regulate the nuclear translocation of NF-κB, and then enhanced the migration of myoblast [12]. Thus, it can be concluded that ECM plays a very important role in the maintenance of the physiological function of satellite cells and the development of skeletal muscle.

With the further understanding of the mechanism of proliferation and differentiation of muscle cells, more and more attention has been paid to the important role of cell niche in development. ECM has also been widely used in the fields of developmental biology, regenerative medicine, and bioengineering due to its important role in regulating cell physiological functions and its unique biological characteristics. Although there have been numerous studies demonstrating the important role of ECM in skeletal muscle development, it is not very systematic. To provide a more comprehensive and systematic concept of the function of the extracellular matrix of skeletal muscle, we provide an overview over the current state of knowledge concerning the structure, composition, function, and application of ECM in skeletal muscle tissue.

## The structure and key components of ECM in skeletal muscle tissue

### Origination and structure of skeletal muscle ECM

Multilayer ECM is a common feature of vertebrates. In 2011, Charvet et al. demonstrated the genesis of ECM during muscle fiber development using zebrafish as a module. They emphasize that the development of myocomma originates from the segmentation period formed by sparse and loosely arranged collagen fibers [13]. During the incubation period of zebrafish, the linkage between actin filaments and sarcolemma was established, followed by the formation of the extracellular basal lamina and the orthogonal arrangement of collagen fibers. Subsequently, fibroblast invaded into the space of myofiber, and a dense network of collagen fibers gradually formed to anchor the myoepithelium or fibroblasts to the basal lamina. An accurate cognition of the structure and genesis of ECM contributes to a deeper understanding of its functions in skeletal muscle development.

To study the structure of ECM visually, researchers have developed a number of tools. Recently, Mayorca-guiliani et al. developed a method to visualize the structure of ECM in detail, called in situ decellularization of tissue (ISDOT). They isolated natural 3D ECM scaffold from tissues with ECM structure and components, and then the structure of ECM could be determined by mapping the protein [14]. In addition, Biela et al. developed a low-molecular fluorescent probe, called COL-F, that penetrates living cells and binds collagen and elastin through non-covalent bonds to image the extracellular matrix without phototoxicity to cells [15].

**Fig. 1:**
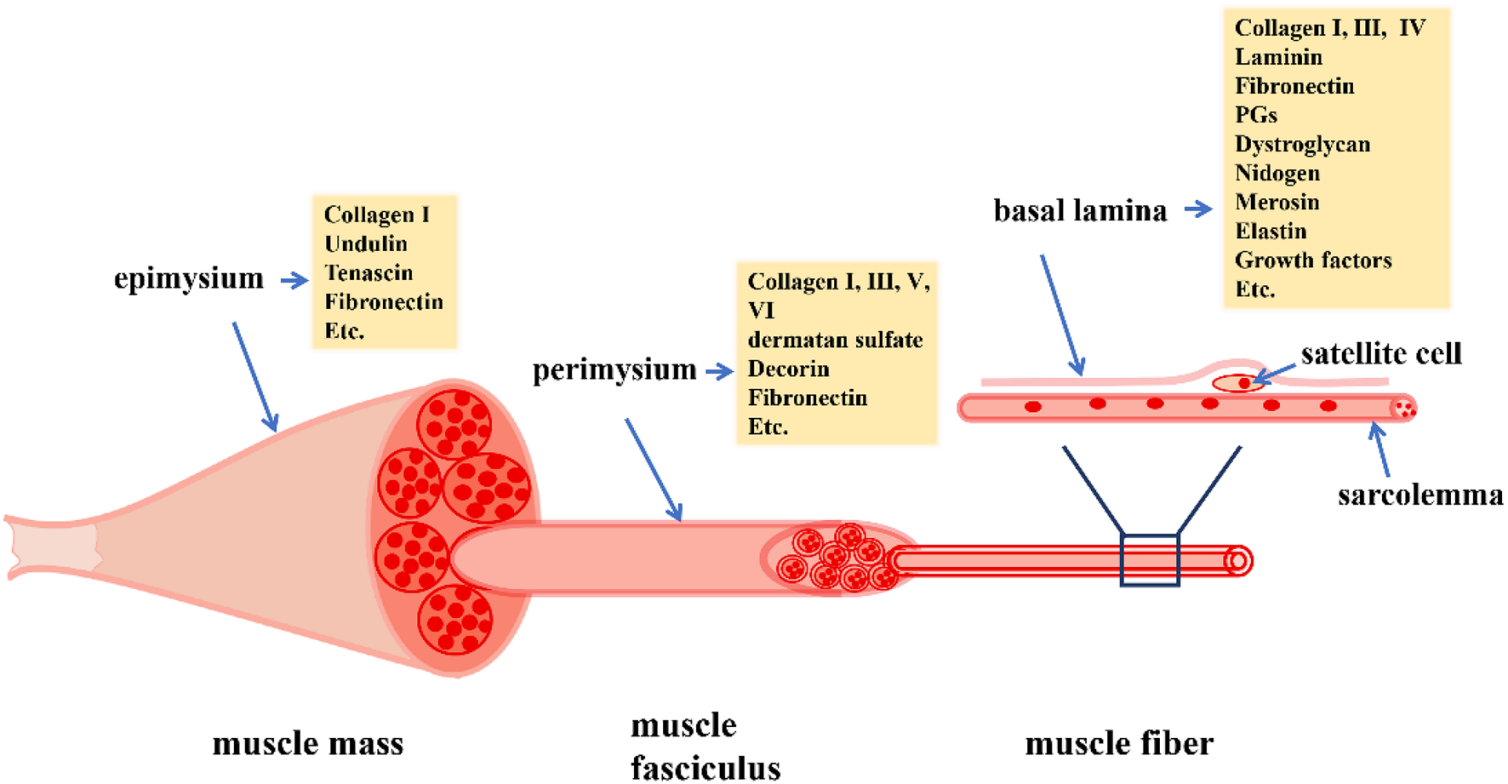
Skeletal muscle ECM three-layer structure diagram

In morphology, the ECM of skeletal muscle tissue can be divided into three independent and interconnected layers: the epimysium is a dense connective tissue that wraps the whole muscle; the perimysium originates from the epimysium and wraps the muscle bundles; the endomysium, also known as basal lamina, is a kind of sophisticated membrane around each muscle fiber [16]. The epimysium contains typeI collagen, undulin, tenascin, and fibronectin etc. [17]; the perimysium contains collagen (I, III, V, and VI etc.), dermatan sulfate, decorin, fibronectin etc. [18–20]; and the endomysium contains type IV collagen, laminin, fibronectin, PGs, growth factor, nidogen etc. (Fig. 1) [21–25].

**Fig. 2:**
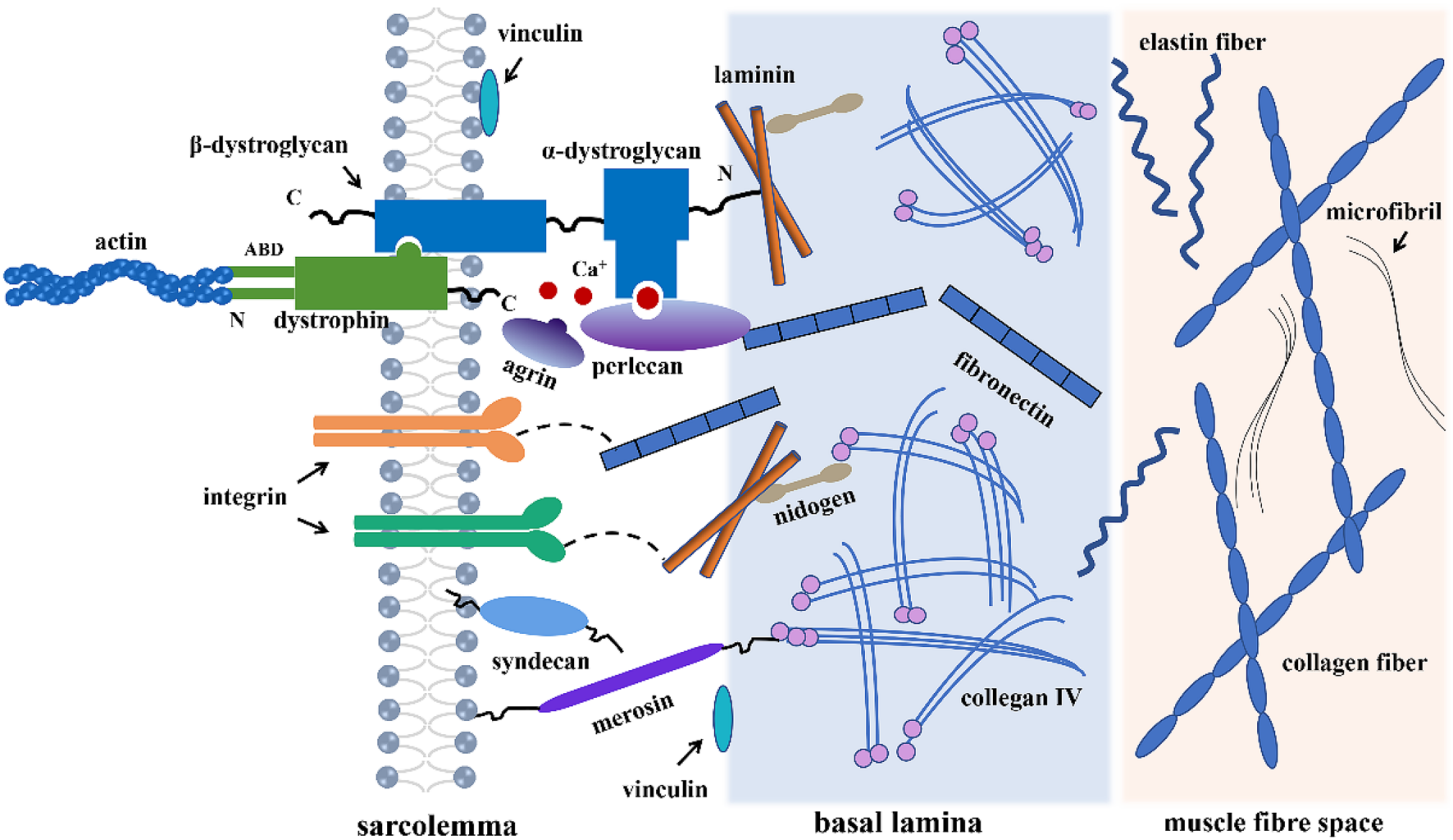
Ultrastructural diagram of basal lamina

The basal lamina is a supramolecular ECM structure, including the inner layer (adjacent to the sarcolemma) and the outer mesh layer (Fig. 2) [26–28]. The integrity of basal lamina is the basis of regeneration of damaged muscle fibers. Li et al. observed the ultrastructure of substrates and found that abnormal basement membrane would lead to limb band muscular dystrophy (LGMD) [29]. During the embryonic skeletal muscle development, Laminin, type IV collagen, and nidogen punctate concentrated in the limb bud of myogenic region, participate in the assembly of basal lamina [25]. Vinculin, perlecan, and dystrophin-glycoprotein complex (DGC) etc. exists between sarcolemma and basal membrane, which are connected by microfilaments [3, 30, 31]. Merosin is a key extracellular matrix protein that forms a mechanical connection between the sarcolemma and collagen. Merosin deficiency can lead to impaired muscle contraction and transmission of force [32]. Plasminogen activator inhibitor-1 also acts as a link between the cell surface and ECM by forming multimolecular compounds containing integrinα5β3 in myogenic cells [33]. The connection between basal lamina is mainly made up of the strut of collagen I, which contain collagen fibers, elastin fibers, and microfibrils, the rest is filled with a polyanionic lattice of unit collagen fibers, microfilaments, and particles [34]. Furthermore, the basal lamina contains a variety of growth factors, which directly participate in the physiological activities of muscle fibers and play an important role in maintaining the physiological functions of skeletal muscle [35–38].

Extracellular matrix is composed of three main proteins, namely, collagen, non-collagen and proteoglycan. Collagen is the largest component of ECM protein in skeletal muscle. In addition, there are receptors and regulators present in extracellular matrix, such as integrin [39] and matrix metalloproteinase (MMP) [40, 41]. Different components of ECM have different distribution and different functions, but all of them are important for maintaining the physiological activities of skeletal muscle.

### Collagen

Collagen is the most abundant component of ECM in skeletal muscle tissue. There are three types of cells that can produce and secrete collagen in mouse skeletal muscle, namely fibroblasts, fibro/adipogenic progenitor cells, and skeletal muscle progenitor cells (MPCs) [42]. Gillies et al. used multiple imaging modalities and quantitative stereology and found that collagen presents large bundles of fibers in the ECM [43]. In addition, collagen can be divided into several subtypes. Type I, III, V, and XI belong to collagen fiber classes that form collagen fiber in the skeletal muscle, and have a good biomechanical performance. Type VI is microfibril protein that form filamentous grid [44]. Type IV and VIII [45] are important components of basal lamina. Type XXII is localized at tissue junctions, and contribute to stabilize the connecting and skeletal muscle tendon adhesion [46]. Researches showed that increased collagen concentration could result in increased skeletal muscle stiffness and decreased mechanical performance but protects smaller muscle fibers from damage [47–49].

TypeI collagen can significantly inhibit myogenic differentiation. Myoblasts and myotubes can synthesize CTGF in the presence of TGFβ and lysophosphatidic acid, thereby inhibiting myoblast over-differentiation by promoting the expression of a variety of ECM components, such as typeI collagen and integrin [50]. Alexakis et al. demonstrated that the expression level of typeI collagen was down-regulated during myoblast differentiation, while the addition of exogenous typeI collagen could significantly inhibit myoblast differentiation [51]. However, studies have shown that typeI collagen could contribute to the proliferation and migration of myoblast [12, 52, 53].

Type IV collagen is one of the main components of basal lamina that can promote the IGF1 mediated migration, differentiation, and fusion of myoblasts, thus promoting the regeneration of skeletal muscle [54]. Col4α1 gene mutation can lead to decreased exocrine secretion of Col4α1, α2, and α3 trimers, resulting in ultrastructural abnormalities and damage of basal lamina, central nucleus concentration, local inflammatory infiltration, and ECM abnormal deposition, thus leading to muscle fiber atrophy [55, 56].

Type VI collagen play an important role in maintaining the physiological function of skeletal muscle. Type VI collagen expressed and secreted by fibroblasts, almost no expression in the muscle cells [57]. However, the enhancer essential for the transcription of Col6α1 gene is induced by the signal factor released by muscle cells, lacking of muscle cell can reduce the deposition of type VI collagen in connective tissue [58]. Moreover, type VI collagen is a key component of satellite cell niche, and the knockout of Col6α1 can reduce the activity and self-renewal ability of satellite cell, thereby weakening the regeneration ability of skeletal muscle [59]. In addition, type VI collagen deficiency could severely damage the components of ECM [60, 61], which cause muscle function disorder, protein function disorder, mitochondrial dysfunction, autophagy dysfunction and microtubule associated protein esterification, leading to premature senility and serious myopathy of skeletal muscle [62–64].

### Laminin

Laminin is located in the basal lamina of muscle fibers [17, 65], which can promote the expression and activation of integrin as well as the proliferation, differentiation, and adhesion of cell [66]. Laminin deficiency will lead to ECM component abnormalities [67], thus affecting the physiological function of skeletal muscle. Goody et al. confirmed that the activation of NAD^+^ -paxillin (PXN) pathway could enhance Laminin organization and maintain the stability of basal lamina, thus contributing to improve the muscular dystrophy phenotype [68].

The canonical expression of different subtypes of laminin protein chains is conducive to regeneration of damaged skeletal muscles. Laminin-1 can maintain the adhesion of muscle fibers on basal lamina, improve muscle performance of mdx mice, relieve degeneration and inflammation of skeletal muscles, shorten regeneration cycle, and promote proliferation and migration of myoblast cells [69–71]. Injection of exogenous Laminin-111 in muscular dystrophy mice can promote the expression of Integrin 7, stabilize the basal lamina, and protect skeletal muscles from sports injury [72]. Moreover, the activation of satellite cells is accompanied by up-regulation expression and deposition of Laminin in the process of regeneration of muscle fiber, and knocking out Laminin-α1 can inhibit the proliferation and self-renewal of satellite cells [73]. In addition, Laminin-α2 mutations can result in loss of function of laminin protein [74] and dissociation of muscle fibers from the basal lamina [75], leading to severe atrophy and abnormal development of muscle fibers, and finally induce the pathological reactions of skeletal muscles [76].

### Fibronectin

Fibronectin is localized in epimysium, perimysium, and endomysium. In addition, fibronectin protein also co-locates with tenascin-C at the tendon junction [17]. Fibronectin is secreted by fibroblasts and activates the integrin proteins through FAK/Src pathway, thereby initiating the peripheral nuclear localization of muscle fibers [77]. The connective tissue hyperplasia of skeletal muscle is mainly composed of fibronectin and collagen [19]. TGFβ can promote the expression of collagen and fibronectin, thereby promoting ECM accumulation and tissue fibrosis [78, 79].

Fibronectin can promote the adhesion and differentiation of myoblasts but inhibit the migration and division [66, 80]. Fibronectin facilitates the fusion and linear alignment of myoblast tubes during myoblast differentiation [81]. Fibronectin deficiency can lead to abnormalities in ECM and muscle tubule formation, leading to skeletal muscle dysfunction [82, 83]. Study showed that the focal adhesion kinase (FAK) pathway can regulate integrin-mediated adhesion and migration of myocytes to fibronectin [84]. Lukjanenko et al. found that fibronectin could be used as the preferred adhesion matrix of satellite cell through ECM Library Screen and Pathway analysis, but due to insufficient adhesion of satellite cell in aging skeletal muscle, integrin-mediated signals could not be transmitted through FAK and P38 /MAPK pathways, resulting in decreased regeneration ability of skeletal muscle, while the reconstruction of fibronectin in aging skeletal muscle could restore its regeneration ability [85]. Moreover, the expression level of fibronectin can affect the remodeling of satellite cell niche, thus affecting the activation and proliferation of satellite cells [86]. Bentzinger et al. showed that fibronectin could bind to Syndecan-4 to promote the expression of Wnt7a, thereby inducing the symmetrical division of satellite cells, and activated satellite cells can also reconstruct niche by autocrine fibronectin [87].

### Dystrophin and dystroglycan

Dystrophin and dystroglycan are important links between cytoskeleton and extracellular matrix, which can maintain the integrity of cell membrane. The N-terminal of dystrophin protein binds to actin through two major actin binding sites (actin binding domain, ABD), and each ABD consists of two calmodulin homologous domains [88]. There are three missense mutations in the ABD structure of skeletal muscle with Duchenne muscular dystrophy (DMD), which leads to the wrong folding of ABD, thus hindering the binding of dystrophin to actin, destroying the connection between muscle fiber membrane and ECM, and leading to pathological reactions [89]. Dystroglycan is localized in the basal side of the outer surface of muscle fiber membrane and involved in connecting the basal lamina and muscle cells [41, 90]. Cullen et al. found that dystroglycan closer to the peripherally of muscle fibers than dystrophin by ultrastructural localization analysis [91]. The last 20 amino acids in the C-terminus of β-dystroglycan bind to the cysteine-rich region of dystrophin, and a chain of dystroglycan extends to the basal lamina to interact with laminin, thus bonding the sarcolemma to the basal lamina [92, 93]. However, phosphorylation of the C-terminal 15th tyrosine of β-dystroglycan can disrupt its binding with dystrophin, thus inducing pathological reactions in skeletal muscle [94].

Dystrophin forms dystrophin-glycoprotein complex (DGC) along with dystroglycan and other proteins, such as dystrobrevin and utrophin [95–97]. DGC is the G protein coupled receptor of laminin in ECM [98]. Abnormal peptide chain [99], glycosylation [100], or binding activity with laminin will cause muscular dystrophy. DGC can also participate in the lateral transmission of force between muscle fibers, while the structure and function disorder of DGC will destroy the lateral transmission, causing instability of power and increasing the sensitivity of muscle fiber to contractile damage [101]. As an important component of DGC, dystrophin is necessary for the formation of stable muscle fiber attachment during skeletal muscle development. Mice lacking dystrophin have severe muscle atrophy, abnormal expression of laminin-α2 chain [102], and impaired vesicle transport [103]. Dystroglycan is a widely glycosylated extracellular protein containing α and β subunits. The inhibition of dystroglycan in skeletal muscle can lead to the damage of cytoskeleton, the decrease of titin, and the increased sensitivity of muscle fibers to contractile damage, thus leading to different types of muscular dystrophy [104]. In addition, α-dystrobrevin (α-DB) as another component of DGC is required for postsynaptic maturation, and a combination of α-DB and DGC provides enhanced postsynaptic stabilization. It follows that DGC is necessary for the physiological function of skeletal muscle.

### Proteoglycan (PGs)

Proteoglycan is an important component in the ECM of skeletal muscle, including glycosaminoglycans, fibromodulin, and heparin sulfate glycosaminoglycan (HSPG) etc. Proteoglycan is involved in connecting the internal cytoskeleton and ECM, while mice with proteoglycan deficiency will exhibit muscle degeneration and muscular dystrophy [105].

Glycosaminoglycans combine with fibrous proteins to improve myoblast proliferation and differentiation [106]. Fibromodulin (FMOD) is a regulator of MSTN, which inhibit the function of Myostatin protein by preventing the correct folding of protein as well as binding to the activin receptor and, thus promoting the recruitment of satellite cells and muscle fiber regeneration [107]. Heparin sulfate proteoglycan, as ECM receptor, is located in the endomysium [24, 108]. For the first time, Brandan et al. identified the presence of HSPGs in basal lamina of mammal skeletal muscle using biochemical indicators, and confirmed that the glycosaminoglycan side chain was only composed of heparin sulfate [109]. HSPGs family contains multiple members, including perlecan, syndecan, glypican etc. Among which perlecan and glypican are mainly connected to ECM structure and syndecan is connected to muscle fiber [110].

## The roles of ECM in skeletal muscle

### Interaction between ECM and muscle cells

ECM is a highly nonlinear elastic material whereas muscle fibers are linear and elastic [111]. ECM serves as a scaffold for cells-matrix interaction that is essential for many physiological activities within the muscle tissue. In skeletal muscle tissue, ECM provides a stable microenvironment that supports the adhesion, migration, proliferation, and differentiation of cell. However, the physiological activity of skeletal muscle also affects the characteristics of ECM. Therefore, the interaction between ECM and muscle cells is beneficial for the adaptation of muscle cells to their microenvironment, thus promoting the development of skeletal muscle.

Studies showed that the supportive and regulatory role of ECM is essential for the formation of muscle tube, and this effect occurs in the early stages of myogenic differentiation [11, 112]. Liu, Yi-Xiao et al. confirmed that ECM could act on skeletal muscle progenitor cells and participate in their proliferation and differentiation through analyzed the protein interaction signals between cells using the Silico Canal-Ligand pairing screen method [9]. Zhang et al. also demonstrated that each kind of cell exhibited better proliferation and differentiation ability in culture media containing ECM extracted from its own original tissue, using decellularize ECM coating [113]. In addition, Stern et al. developed a method to extract ECM from adult rat leg muscles and use it as a surface coating to culture myoblasts, demonstrating that myoblasts cultured on ECM extract have enhanced proliferation and differentiation ability [114]. In the absence of ECM, the expression of myogenic differentiation factors is insufficient to successfully initiate skeletal muscle differentiation. Osses et al. showed that inhibiting of the deposition and assembly of ECM components can effectively inhibit myogenesis, but doesn’t affect the expression of MyoD, Myogenin, and MEF2A, while the addition of exogenous ECM can reverse these effects [115].

Likewise, the physical activity of muscle cells also affects the composition of the ECM. Kaasik et al. have shown that muscular unloading and reloading could influence the composition of the ECM. Unloading could down-regulate the expression level of typeI, III, and IV collagen, while reloading could strengthen the expression of collagen, MMP-2, and tissue inhibitor of metalloproteinase-2 (TIMP2) in the fast muscle fibers [116]. In serum-free medium, myoblasts can rapidly secrete and organize their own matrix proteins to create a local ECM microenvironment to support its survival [117]. In addition, satellite cells can negatively regulate the expression of ECM-related genes in fibroblasts in vitro, and the absence of satellite cells in skeletal muscle will lead to excessive accumulation of ECM and increase of muscle fibrosis [118, 119].

These studies indicated that myogenic differentiation can regulate muscle microenvironment, which in turn regulates the cell behavior during skeletal muscle development. The ultimate purpose of the interaction between cells and their niche is to better “serve” the development of tissues. In this process, the cell is the functional actor and the extracellular matrix acts as a regulation factor.

### ECM in physiological function of muscle stem cells

Skeletal muscle stem cells, also known as satellite cells, are activated when skeletal muscle development or damaged, and subsequently proliferate, differentiate, and fuse to form new muscle fibers. In the development of skeletal muscle, ECM provides a stable microenvironment for the migration, adhesion, proliferation, and differentiation of satellite cells. Overexpression of ECM proteins can lead to alteration in niche of satellite cells and weaken the differentiation ability of satellite cells, thus affecting the development of skeletal muscle [9, 59, 120]. In addition, ECM remodeling is a key step in the complete process of satellite cells from activation to proliferation and self-renewal. Study showed that the activation of satellite cells is accompanied by local remodeling of ECM, resulting in up-regulation expression and deposition of laminin-α1 and laminin-α5 in the basal lamina. MMPs can activate the remodeling of ECM and initiate the activation of satellite cells. Inhibiting MMPs can effectively inhibit the deposition of laminin in satellite cell niche and prevent the activation, differentiation and self-renewal of satellite cells [73]. Moreover, Moyle et al. confirmed that the synergistic effect of ECM stiffness and WNT7 could regulate the symmetrical division of satellite cells, thus affecting the fate of satellite cells [121]. Excessive accumulation of ECM in the microenvironment of aging skeletal muscle resulted in increased stiffness, thereby inhibiting the myogenic differentiation ability of satellite cells [8].

However, different components of ECM have different effects on myoblast behavior. Studies showed that the promoting effect on proliferation and differentiation of satellite cells of complete ECM and laminin were better than collagen and fibronectin, while fibronectin and laminin can improve the adhesion and differentiation ability of satellite cells but inhibit the proliferation and migration of cells [66, 80]. Moreover, Chaturvedi et al. showed that complete ECM and fibronectin could induce the formation of ordered myotubes, while the addition of collagen led to disordered myotube sequence [117]. In addition, the expression of ECM component required to maintain satellite cell niche in skeletal muscle of young mice was upregulated compared with that of aging mice [122].

### ECM in regeneration of muscle

Intact ECM can support regeneration of muscle fibers in damaged skeletal muscles. Zhang et al. produced d-ECM from porcine skeletal muscle, liver and kidney, and modified with heparin hyaluronic acid hydrogel (ECM-HA-HP), studies have shown that satellite cells show stronger ability of proliferation, differentiation, and fusion on muscle ECM-HA-HP (mECM-HA-HP) substrate, which can be used for cell therapy of skeletal muscle dysfunction [123]. Also note that the successful regeneration of damaged muscle fibers begins with the migration and activation of satellite cells. Webster et al. found a residual extracellular matrix, called "Ghostfibers", in the impaired skeletal muscle fibers using 3D time-lapse intravital imaging technology, which regulates the behavior of skeletal muscle progenitor cells during the process of regeneration. Their study showed that satellite cells divided and migrated along the longitudinal axis of the "Ghostfibers" after activation, and changing the direction of the "Ghostfibers" could change the migration path and cleavage plane of myogenic progenitors, thus disrupting the regeneration process [124].

In the early stage of muscle fiber injury, ECM hyperplasia results in increased skeletal muscle tissue stiffness, and this orderly deadhesion, and fibrosis is designed to protect skeletal muscle from further damage [8, 87, 125]. With the differentiation of satellite cells, ECM is remodeled (including changes in growth factors, glycosaminoglycan, and basement membrane structural proteins, etc.), accompanied by up-regulation of adhesion protein expression [35, 125–127]. In skeletal muscle injury or myopathy, genes associated with ECM remodeling are up-regulated [128]. Moreover, activation of satellite cells induces local remodeling of ECM to repair the damaged basal lamina [73]. Furthermore, ECM releases cytokines that promote the proliferation of myogenic progenitor cells, such as FGF2, HGF, and SDF-1, and then promote the regeneration of myofiber by inducing the transcription of MeF2, MyoD, Myf5, and Myogenin in progenitor cells [36]. Therefore, ECM remodeling is an important link in skeletal muscle regeneration.

According to these studies, we can conclude that ECM, as an important component of muscle fiber niche, plays an important role in muscle fiber regeneration and skeletal muscle development. ECM component proteins are secreted by a variety of cells surrounding muscle fibers, such as fibroblasts, endothelial cells, and skeletal muscle connective tissue cells [129–131]. Therefore, studying cell-to-cell interactions is helpful for us to understand the regulatory mechanism of satellite cell activation, proliferation, and differentiation.

### ECM in signal transduction of neuromuscular junction

ECM components are essential for the development of neuromuscular junction (NMJ). Study showed that ECM proteins could promote the activity of acetylcholinesterase [11]. In addition, local ECM environment can regulate the synaptogenesis in the process of synaptic induction. At the NMJ of skeletal muscle, the basal lamina crosses the synaptic cleft, where laminin is involved in regulating synaptic localization and signaling [23]. Recent study has shown that ECM-induced PLSs (Podosome-like structures) regulated the formation and reconstruction of acetylcholine receptor (AchR) clusters by regulating local ECM degradation, and PLSs can also degrade ECM by mediating the transport and insertion of MT1-MMP matrix metalloproteinase to the surface of the AchR cluster [132].

In addition, various proteins in the ECM, such as collagen [133], integrin [134], and dystrophin [103], participate in the development and maturation of NMJ. Sigoillot et al. showed that ColQ could regulate the development and maturation of postsynaptic domains through regulating the expression of synaptic genes, while ColQ deficiency will lead to the up-regulation of the five subunits of nicotinyl acetylcholine receptor, resulting in the mixture of mature and immature AchR in the neuromuscular junction [135]. Moreover, type VIII collagen deficiency can lead to the imperfect adhesion between presynaptic and postsynaptic membrane, resulting in synaptic structure defects, and thus affect the signal transduction and acetylcholine receptor cluster development [136]. Furthermore, study also showed that integrin α3 could be involved in the localization of active zone (AZ) components and the effective release of synaptic vesicles as well as the deposition of synaptic basement membrane [137].

The integrity of the neuromuscular junction and the transduction of synaptic signals are the keys to the motor function of skeletal muscle, while the abnormal deposition of ECM protein will lead to the disorder of the connection between motor neurons and muscle fibers [137, 138]. It can be seen that the composition of ECM is closely related to the motor function of skeletal muscle. Therefore, researchers should pay more attention to the expression of ECM components when studying the exercise physiology of skeletal muscle in the future.

### ECM in the transmission of force in skeletal muscle

ECM can exert transverse stress on fibers and have axial strain [139]. If the connection between ECM and muscle cells is insufficient, muscle fibers will lack mechanical support and the force transmission pathway in which ECM is involved will be damaged, resulting in the deformation of muscle fibers beyond the physiological limit [140]. Dystrophin-glycoprotein complex (DGC) is an important linkage between muscle fiber cytoskeleton and extracellular matrix, which is involved in the transverse transmission of muscle fiber power. In the process of muscle fiber contraction, the force generated by skeletal muscle of young mice does not decrease when transversely transferred from fiber to fiber, while due to the disorder of structure and function of DGC in muscular dystrophy or aging mice, the transverse transmission of force is destructed, which increases the sensitivity of muscle fibers to contractile injury [101].

After skeletal muscle injury, ECM can improve muscle function to a certain extent by regulating the force transmission at the injured site rather than relying on skeletal muscle regeneration [141]. The stiffness of ECM affects the mechanical force transferred at the end of muscle fibers. Study showed that the stiffness and fiber arrangement of ECM were important factors affecting the force transfer during muscle contraction, which is of great significance in the application of engineering skeletal muscle [142]. Aging [8], tendon resection [143], and myopathy [47, 144] etc. can all lead to ECM hyperplasia and stiffness increase. Stearns-Reider et al. quantitatively analyzed the topological structure of ECM and the mechanical properties of muscles, showing that with age, collagen bending decreases, extracellular matrix stiffness increases, and the mechanical properties of skeletal muscle decreases [7]. Therefore, any abnormal state of skeletal muscle will affect its mechanical properties, while normal assembly of ECM will improve muscle weakness to some extent.

In addition, ECM is also the major contributor to the passive tension of skeletal muscle [145]. Studies showed that the fiber network of ECM can be normalization and densification in the direction of force through stress-induced tension, which is conducive to muscle fiber contraction and cell migration [146, 147]. Marcucci et al. obtained the passive tension value of ECM fiber by subtracting the passive tension of muscle bundle and fiber, and then compared it with the passive tension of muscle fiber, proving that the modulus and tensile carrying capacity of ECM are higher than that of muscle fiber [148]. ECM hyperplasia can lead to the increase of stiffness and passive tension in skeletal muscles [149]. Azizi et al. have studied the mechanical interaction between contractive muscles and ECM. The results showed that with the increase of ECM content in skeletal muscle, the ability of muscle to expand radially was impaired, which in turn limited the muscle shortening and increased the passive tension in the muscle [150]. Resistance training can reduce tissue fibrosis and induce ECM remodeling, thus improving the mechanical properties of skeletal muscle [151–153].

Main conclusion is that excessive accumulation of extracellular matrix can significantly impair the mechanical properties of skeletal muscle, including active and passive tension. Therefore, the remodeling of extracellular matrix and the correct expression of each component are of great significance in the clinical treatment of muscle weakness.

### ECM in muscle pathophysiology

Although the characteristics, components, and function of the ECM vary in different tissues, it is common that any deficiency in ECM properties can cause pathophysiological responses, such as chondrodysplasia [154], Ehlers-Danlos syndrome [155], and myodystrophy. In skeletal muscle diseases, the degenerative changes of muscle fibers are characterized by the gradual replacement of individual muscle fibers by connective tissue. The process involves the exfoliation of peripheral cytoplasm into the endomysium cavity, resulting in muscle fiber contraction and collagen fiber fragmentation, and eventually the hollow basement membrane sheath is surrounded by abundant extracellular matrix [156]. The myopathy phenotypes caused by defects in different components of ECM are also different. COLQ deficiency leads to abnormal development of neuromuscular junctions in adult mice, resulting in a myoatrophy phenotype [135]. Type VI collagen defects can lead to premature aging and dysfunction of skeletal muscle and the morphological change of tendon [60, 62]. In addition, abnormal expression of laminin, fibronectin and proteoglycan can lead to severe myopathic phenotypes in skeletal muscle, such as DMD syndrome. Therefore, the study on the composition and characteristics of ECM has guiding significance for the clinical treatment of ECM related diseases.

## Application of ECM in biomedical and engineering

ECM is necessary for tissue development, so it has a good application prospect. Decellularize ECM (dECM), which is derived from in vivo, is widely used in the field of bioengineering and regenerative medicine because of its excellent histocompatibility and biological properties. It can be used as a biological scaffold to promote the formation of functional tissues. Kao et al. prepared pig bladder matrix hydrogels using Sodium Dodecyl Sulfate Decellularization Method, and the results showed that the SDS Decellularization Method provides a more stable and safer access to the Decellularization bladder matrix due to reduced immunogenicity and can be used as a potential candidate scaffold for tissue remodeling [157]. Nikniaz et al. compared different methods of tissue decellularization. The results show that compared with other acellular methods, SDS-Triton-Ammonium treatment group has lower DNA residue and better biocompatibility [158].

In recent years, researchers have developed dECM active materials for clinical treatment using bioengineering techniques. Trevisan et al. constructed mouse decellularized diaphragm ECM, which can promote the activation, proliferation and differentiation of skeletal muscle progenitor cells to form a powerful three-dimensional skeletal muscle structure, providing a promising tool for clinical application of diaphragm regeneration in the future [159]. Lee et al. used skeletal muscle-derived dECM and IGF1 to develop a decellularized muscle-specific scaffold system, which can better promote cell proliferation and differentiation, thus supporting in situ regeneration of muscle tissue [160]. Kim et al. collected the decellularized ECM from porcine skeletal muscle by using the decellularize technology, then used 3D printing technology to construct the dECM-based structure that laden myoblast cells to form a functional structure with skeletal muscle tissue characteristics, which can be used for drug screening and in vitro chip development [161]. Zhu et al. designed ECM scaffolds with parallel microchannels, which can closely observe the activity of cells in vitro and contribute to the infiltration and angiogenesis of transplanted cells in vivo, and can be applied to the development of inducible biomaterials and regenerative medicine [162].

ECM materials can support cell attachment and proliferation in vitro, and have good anti-inflammatory and immunosuppressive properties in vivo, which can improve the success rate of cell transplantation [163]. In the clinical treatment of skeletal muscle disease, the degradation products of ECM biological scaffolds can also promote the alternate activation and polarization construction of M2 macrophages, thus promoting the migration and myogenesis of skeletal muscle progenitor cells [164, 165]. The application of ECM in the clinical treatment of diseases is the result of the comprehensive application of multidisciplinary such as cell biology, bioengineering, and regenerative medicine. Although the technology has become increasingly developed, there are still many aspects to be improved. Therefore, I think the future research direction should focus on the accuracy of the effect, the operability of the method, and the control of the cost.

## Discussion

ECM is a complex and sophisticated structure whose components are synthesized and secreted by many types of cells. In this paper, the characteristics and functions of ECM in skeletal muscle tissue are discussed in detail. The cytoskeleton forms a close connection with ECM through DGC, laminin, and proteoglycan, etc. ECM can not only maintain skeletal muscle morphology and contraction as a scaffold, but also regulate various physiological functions of skeletal muscle, such as signal transmission of motor neuron, glucose metabolism, and regeneration after injury. Furthermore, decellularized ECM as biomaterials is widely used in bioengineering and regenerative medicine because of its unique and superior biological characteristics. Therefore, the in-depth study of ECM is beneficial for researchers to further explore the mechanism of skeletal muscle development, and provide new insights for clinical treatment of skeletal muscle diseases and the development of biological materials.

However, ECM does not function independently and also requires the involvement of multiple cytokines, such as Integrin, MMPs, and TGFβ. Integrin, as receptor of extracellular matrix proteins, coordinate with extracellular matrix to regulate the adhesion [166], proliferation [73], migration [84], and differentiation [167] of myoblasts, as well as the force transmission of muscle fibers [168, 169] and development of synapses [134, 137]. MMPs are important factors that induce ECM remodeling. Skeletal muscle injury [170] or exercise [171] will cause changes in the expression level of MMP protein, and thus participate in the regulation of muscle fiber repair and hypertrophy by regulating the remodeling of ECM. TGFβ promotes ECM deposition by promoting the expression of ECM-related proteins. Currently, some drugs are widely used to inhibit the excessive accumulation of ECM by inhibiting TGFβ expression, so as to achieve the purpose of myopathy treatment [172–174]. Accordingly, it may be more beneficial for researchers to explore new molecular mechanisms by considering the interactions between cells and the regulatory network upstream and downstream of ECM.

## Data Availability

Not applicable.
